# Gene expression in “young adult type” breast cancer: a retrospective analysis

**DOI:** 10.18632/oncotarget.4051

**Published:** 2015-05-09

**Authors:** Rebecca H. Johnson, Pingzhao Hu, Cheng Fan, Carey K. Anders

**Affiliations:** ^1^ Seattle Children's Hospital/University of Washington, Seattle, WA, USA; ^2^ Department of Biochemistry and Medical Genetics, University of Manitoba, Winnipeg, Canada; ^3^ University of North Carolina, Lineberger Comprehensive Cancer Center, Chapel Hill, NC, USA; ^4^ Department of Medicine, Division of Hematology-Oncology, University of North Carolina, Chapel Hill, NC, USA

**Keywords:** breast cancer, young, premenopausal, gene expression, survival

## Abstract

Background: Young women with breast cancer experience inferior outcome and commonly manifest aggressive biological subtypes. Data is controversial regarding biological differences between breast tumors in young (diagnosed at <40 years of age) *versus* older women. We hypothesize there may be age-related expression differences in key genes for proliferation, invasion and metastasis within and across breast cancer subtypes, and that these differences correlate with outcome.

Methods: Using clinically-annotated gene expression data from 778 breast tumors from three public databases, we compared clinico-pathologic characteristics, mRNA expression of 17 selected genes, and outcome, as a function of age (< 40 years *vs.* ≥ 40 years).

Results: 14 of 17 genes were differentially expressed in tumors of young *vs.* older women, 4 of which persisted after correction for subtype and grade (p ≤0.05). *BUB1, KRT5*, and *MYCN* were overexpressed and *CXCL2* underexpressed in young women. In multivariate analysis, overexpression of cytokeratin genes predicted inferior DFS only for young women. Overexpression of *ANGPTL4* strongly predicted inferior DFS in basal but not HER2-enriched tumors in young women. Overexpression of cytokeratin genes and MYBL2 and low SNAI1 expression correlated with inferior DFS in HER2-enriched tumors in younger women. Kaplan-Meier analysis within the basal and HER2-enriched subgroups showed that overexpression of cytokeratin genes was associated with inferior DFS for young, but not older women.

Conclusions: This preliminary study reveals age- and subtype-related differences in expression of key breast cancer genes for proliferation, invasion and metastasis, which correlate with prognostic differences in young women and suggest targeted therapies.

## INTRODUCTION

Breast cancer is the most common malignancy in young women aged 15-39 years, and young age is an independent risk factor for death from breast cancer [1]. Young women tend to present with higher grade, biologically-aggressive tumors (i.e. basal and HER2-enriched subtypes) compared to older women [2]. Women under 40 years of age with early stage breast cancer are 40% more likely to die of their disease than older counterparts [3]. While clinicopathologic differences point to underlying biologic differences between the breast tumors arising in younger versus older women, prior studies have yet to document age-related changes in global gene expression beyond those attributable to increased frequency of aggressive subtypes in younger patients [4].

There is currently limited data to explain why a higher percentage of younger versus older women develop biologically aggressive breast cancer subtypes, nor why young women with early stage disease have disproportionately higher mortality compared to older women. In this study we selected a candidate gene approach to address this question, analyzing the expression of well-known breast cancer genes with strong potential for prognostic significance as a function of age. A notable study describes a 5-gene classifier that holds prognostic significance [5]. This classifier takes into account protein expression of epithelial growth factor receptor (EGFR) and cytokeratin (CK) 5/6 by immunohistochemistry, in addition to expression of estrogen receptor (ER), progesterone receptor (PR) and HER2 (human epidermal growth factor receptor 2). Patients with triple negative breast cancer in the “core basal” subgroup (whose tumors lacked expression of ER, PR and HER2, yet expressed EGFR and/or CK 5/6) had inferior outcome following anthracycline-based therapy regimens as compared to patients whose tumors lacked expression of all 5 biomarkers. The “core basal” subtype was more commonly seen among younger women under 40 years of age as compared to older women. Specifically, 18% of breast cancers were “core basal” (71/380) among patients aged ≤40 compared to 7% (265/366) among patients aged >40 [6] [7]. Thus, we hypothesized that there may be age-specific differential expression of key genes relating to breast cancer proliferation, invasion or metastases, including CK 5/6, EGFR, and others, across and within breast cancer subtypes, and that these differences may hold prognostic importance.

## RESULTS

### Age-specific clinical characteristics

Of the 778 patients included in this analysis, 13% (*n* = 103) were aged < 40 years (24-39 years of age, with median age 36) while 87% (*n* = 675) were aged 40 years or older (40-93 years of age, with median age 52) (Table 1). A higher proportion of younger women were diagnosed with HER2-enriched and basal breast cancers when compared to older women (23.3% versus 17.2% and 41.8% versus 23%, respectively). Conversely, older were more likely to be diagnosed with Luminal A breast tumors when compared to younger women (37.6% versus 15.5%). Relative to Luminal A breast tumors, younger women were more likely to be diagnosed with Luminal B (Odds Ratio [OR] = 2.11, *p* = 0.03), HER2-enriched tumors (OR = 3.27, *p* = 0.0007), and basal (OR 4.39, *p* = 0.0000005) breast cancer. In addition, consistent with previous reports [15], young women were also more likely than older women to be diagnosed with grade 3 tumors (OR = 4.05, *p* = 0.0002), while they were less likely to be diagnosed with ER positive as compared to ER negative breast tumors (OR = 0.51, *p* = 0.003). More older women received endocrine therapy (with or without chemotherapy), likely a result of a greater proportion of older (*vs.* young) women being diagnosed with endocrine sensitive breast cancer. Rates of receipt of chemotherapy as a single modality of treatment were similar between age groups (*p* = 0.23).

**Table 1:** Clinical information for young and older patients

Characteristic	All (n=778)		Younger (<40, n=103) 24-39 years of age		Older (>=40, n=675) 40-93 years of age		Odds Ratio	*P*	95% CI
	No.	%	No.	%	No.	%			
Subtype (PAM50)									
Lum A	270	34.7	16	15.5	254	37.6	1.00	NA	NA
Lum B	170	21.9	20	19.4	150	22.2	2.11	0.03	1.01-4.51
HER2	140	18.0	24	23.3	116	17.2	3.27	0.0007	1.60-6.86
Basal	198	25.4	43	41.8	155	23.0	4.39	0.0000005	2.33-8.65
Estrogen* Receptor									
Negative	234	30.6	45	44.1	189	28.5	1.0	NA	NA
Positive	530	69.4	57	55.9	473	71.5	0.51	0.003	0.32 – 0.80
Tumor Size*									
<=2cm	300	38.9	42	41.2	258	38.5	1.0	NA	NA
>2cm	472	61.1	60	58.8	412	61.5	0.89	0.66	0.57 – 1.40
Grade*									
1	126	16.5	7	6.9	119	17.9	1.0	NA	NA
2	280	36.6	25	24.8	255	38.5	1.66	0.32	0.68 – 4.69
3	358	46.9	69	68.3	289	43.6	4.05	0.0002	1.79 – 10.75
Nodal Status*									
Negative	390	51.0	49	48.0	341	51.5	1.0	NA	NA
Positive	374	49.0	53	52.0	321	48.5	1.15	0.53	0.74 – 1.78
Treatment**									
Local only or Nothing***	178	31.1	40	44.9	138	28.5	1.0	NA	
Chemo only	176	30.7	30	33.7	146	30.2	0.71	0.23	0.40 – 1.24
Chemo and Tam /Hormone	101	17.6	12	13.5	89	18.4	0.47	0.037	0.21 – 0.97
Tam/Hormone only	66	11.5	1	1.1	65	13.4	0.05	0.000026	0 – 0.33

The combined data set includes 778 arrays/patients. Fisher's exact test was used to evaluate the proportion differences of young (< 40 years old) and older (>=40 years old) patients with each of the PAM50 tumor subtypes and other clinical variables. The groups with NA in P and 95% CI columns are baseline group.

* The variables have missing values Abbreviations: NA: not available; PAM: prediction analysis of microarray; Lum: Luminal; HER2: human epidermal growth factor receptor 2; CI: confidence interval.

** Based on only data from two studies: NKI295 and GSE20624, which have this information. The combined data has 89 young and 484 older patients, respectively. 6 of the 89 and 46 of the 484 patient missed the treatment information, respectively.

*** Local control includes radiation therapy.

### Age-specific differences in disease-free survival

**Figure 1:**
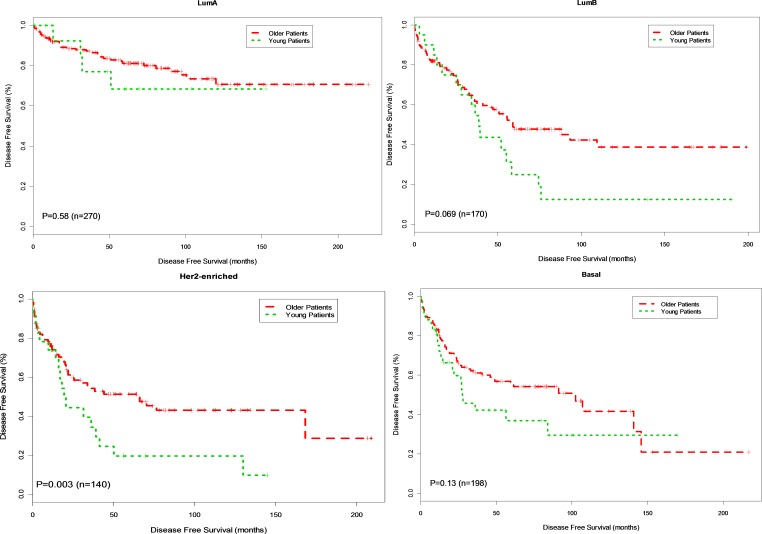
Kaplan-Meier DFS Curves for Young and Older Patients by Subtype Breast tumor samples were classified into young patients (age < 40 years) and older patients (age>=40 years). Disease free survival (DFS) analysis between young and older patients was performed for Basal, Her2-enriched, Luminal B and Luminal A, respectively.

Consistent with previous reports [15], young patients with breast cancer in this dataset experienced inferior disease free survival compared to older patients (Hazard Ratio [HR] = 1.91, *p* < 0.00001) and 10-year survival of 35.0% *vs.* 60.1% for young vs. old patients, respectively. Within subtypes, survival for young versus older patients with Luminal A breast cancer was not significantly different (Figure 1; HR 1.33, *p* = 0.58) and 10-year survival is 62.5% versus 73.2% for young vs. old patients, respectively. There was trend toward inferior survival in young patients with the basal (Figure 1; HR = 1.46, *p* = 0.13; 10-year survival is 46.5% versus 54.8%) and Luminal B breast cancer (Figure 1; HR=1.67, *p* = 0.069; 10.0% versus 51.3%) compared to older patients. Young patients with HER2-enriched breast cancer had significantly inferior outcome compared to older patients with HER2-enriched breast cancer (Figure 1; HR = 1.83, *p* = 0.003) and 10-year survival is 16.7% versus 50.0% for young vs. older patients, respectively.

### Analysis of single gene expression by age

We analyzed the expression of 17 genes key to breast cancer proliferation, invasion, and metastasis as a function of age (Table 2). Before adjustment for subtype and tumor grade, 14 of the 17 genes were differentially expressed in young compared to older patients (*p* < 0.05). Thirteen of the fourteen genes were overexpressed, while one gene (*CXCL2*) showed decreased expression in young *versus* older women (Table 3, Univariate Model). Correction for tumor subtype and grade was performed in a multivariate regression model and 4 genes remained differentially expressed in young versus older women (Table 3, Multivariate Model) (*p* < 0.05). Three of the four genes were overexpressed in breast tumors arising in younger women compared to older women: *BUB1* (Fold Change 1.67, *p* = 0.029), *KRT5* (Fold Change 1.56, *p* = 0.002), and *MYCN* (Fold Change = 1.16, *p* = 0.027). *EYA2* had borderline significance for overexpression in young versus older women (Fold change 1.22, *p* = 0.059). One gene, *CXCL2*, showed decreased expression in the breast tumors arising in young versus older women (Fold Change -1.35, *p* = 0.052).

**Table 2:** Selected 17 candidate genes in relation to breast cancer proliferation, metastasis and outcome

Gene name	Gene ID	Function	Reference
ADM	133	Involved in hypoxia response in lymphatic cells. High expression associated with distant metastatic disease and poor outcome.	[26]
ANGPTL4	51129	Expression is induced by inflammation and tissue hypoxia in endothelial cells. Induces angiogenesis, facilitates cell migration. Mediates vascular metastasis to lungs. High expression associated with distant metastatic disease and poor outcome. Potential druggable target.	[18] [21] [23] [26]
AURKA	6790	Overexpression associated with cell proliferation. Located on Chromosome 20q13, a region frequently amplified/overexpressed in high grade, node negative breast tumors with poor outcome. Homozygotes for some minor SNP alleles have inferior survival and increased frequency of receptor negative tumors	[27][28]
BUB1	699	Associated with poor prognosis in ER-positive/HER2 negative breast cancer. Mitotic checkpoint gene. Overexpression is associated with cell proliferation, may be a marker for chromosomal instability, and predicts poor outcome in breast and other cancers.	[28][29][30][31]
CXCL12	6387	High expression promotes tumor proliferation, migration, invasion. High expression associated with distant metastatic disease	[26] [32]
KRT5	3852	Overexpression of cytokeratin 5/6 associated with inferior outcome in basal subtype	[5]
KRT6A	3853	Overexpression of cytokeratin 5/6 associated with inferior outcome in “core basal” subtype	[5]
KRT6B	3854	Overexpression of cytokeratin 5/6 associated with inferior outcome in “core basal” subtype	[5]
EGFR	1956	Overexpression associated with inferior outcome in “core basal” subtype	[5]
MYBL2	4605	Involved in cell proliferation and survival. Overexpression common in high grade, node negative breast cancer and associated with poor response to therapy and inferior outcome. Gene that appears most often in microarray classifiers.	[27][33]
MYCN	10397	Overexpression associated proliferation, high grade tumors and with inferior outcome	[28] [34]
SNAI1	4613	Promoter of mammary tumor recurrence, expression associated with basal subtype	[26]
UGT8	6615	Overexpression correlates with increased risk of lung metastases in node-negative breast cancer	[35]
VEGFA	7368	Involved in hypoxia response in endothelial cells. High expression associated with distant metastatic disease and poor outcome. Induces angiogenesis. Potential druggable target.	[26]
SIX1	7422	Implicated in Epithelial/Mesenchymal Transition leading to metastasis. Overexpression correlates with advanced disease	[36]
EYA2	6495	Overexpression of EYA2, together with SIX1, predicts poor outcome. Involved in TGF-beta signaling	[36]

**Table 3:** Differential expression analysis between young and older patients

Gene name	FC	Univariate Model				Multivariate Model			
		Beta	SE	*P* value	Adjusted P value	Beta	SE	*P* value	Adjusted *P* value
ADM	1.28	0.49	0.20	0.010	0.014	−0.01	0.18	0.959	0.959
ANGPTL4	1.11	0.17	0.20	0.390	0.390	−0.10	0.20	0.619	0.702
AURKA	1.35	0.55	0.13	<0.001	<0.001	0.12	0.09	0.202	0.429
**BUB1**	**1.67**	**0.77**	**0.15**	**<0.001**	**<0.001**	**0.24**	**0.11**	**0.029**	**0.164**
**CXCL12**	**-1.35**	**-0.54**	**0.15**	**<0.001**	**0.001**	**-0.29**	**0.15**	**0.052**	**0.201**
**KRT5**	**1.56**	**0.91**	**0.24**	**<0.001**	**<0.001**	**0.62**	**0.20**	**0.002**	***0.034***
KRT6A	1.36	0.53	0.16	0.001	0.002	0.18	0.15	0.241	0.455
KRT6B	1.35	0.62	0.19	0.001	0.002	0.22	0.16	0.172	0.418
EGFR	1.07	0.10	0.09	0.290	0.308	−0.06	0.08	0.456	0.613
MYBL2	1.39	0.56	0.12	<0.001	<0.001	0.12	0.09	0.158	0.418
NDRG1	1.16	0.34	0.17	0.040	0.052	−0.08	0.16	0.604	0.702
**MYCN**	**1.16**	**0.15**	**0.05**	**0.006**	**0.009**	**0.12**	**0.05**	**0.027**	**0.164**
SNAI1	1.15	0.21	0.10	0.050	0.061	0.08	0.11	0.441	0.613
UGT8	1.29	0.39	0.13	0.004	0.006	0.04	0.11	0.737	0.783
VEGFA	1.22	0.43	0.13	0.001	0.002	0.13	0.12	0.281	0.478
SIX1	1.26	0.33	0.26	0.206	0.233	0.19	0.26	0.469	0.613
EYA2	1.22	0.37	0.12	0.003	0.005	0.24	0.13	0.059	0.201

Age-specific differences in single-gene mRNA expression values were tested using linear regression model at both univariate and multivariate levels. The multivariate model adjusted for tumor subtype and grade. The estimate (Beta), standard error (SE) and P value are for age term in the regression models. Fold change (FC) is defined as the ratio of mean expression in young patients compared to that in older patients. The bold genes are significant at nominal 0.05 significance level and the bold italic genes are significant at 0.05 adjusted significance level in multivariate model.

### Association between gene expression and DFS in univariate and multivariate models

Initially, the association between DFS and gene expression of the 17 selected genes was performed for young and older patients, respectively (Table 4 and Supp. Table 1). In the young group, significant associations were found between DFS and the following genes: *ADM, ANGPTL4, AURKA, KRT6A, EGFR, MYBL2* and *VEGFA (* Table 4, Univariate Model, all *p* < 0.05). In the older group, mRNA expression levels of *ADM, ANGPTL4, AURKA, EGFR, MYBL2,* and *VEGFA* were also associated with DFS; however, expression of *KRT6A* was not associated with outcome in women over age 40 (Supplemental Table 1, Univariate Model).

Given that breast cancer is a heterogenous disease, the analysis was performed using a multivariate model correcting for breast cancer subtype and grade. In the younger patients, increased expression of the following genes was associated with inferior DFS: *ANGPTL4, KRT5, KRT6A, KRT6B, MYBL2 and VEGFA* (Table 4, Multivariate Model, all *p* < 0.05). Similarly, in the older patients, *ANGPTL4, MYBL2* and *VEGFA,* all maintained significance after correcting for subtype and tumor grade in multivariate analysis (Supplemental Table 1, Multivariate Model). Importantly and in contrast to observations among younger women, expression of *KRT5, KRT6A, KRT6B* were not associated with outcome among older patients (Supplemental Table 1, Multivariate Model). In the more stringent analysis of this data correcting for multiple gene comparisons, interestingly *ANGPL4* and *VEGFA* maintained significance in the younger, but not the older group of patients (adjusted *p* < 0.05).

**Table 4:** Association between expression levels and DFS in all subtypes (Young group)

Gene Name	Univariate Model				Multivariate Model			
	Hazard Ratio	95% CI	*P* value	Adjusted *P* value	Hazard Ratio	95% CI	*P* value	Adjusted *P* value
ADM	1.18	1.06-0.32	0.003	0.012	1.12	0.98-1.27	0.087	0.185
***ANGPTL4***	***1.38***	***1.21-0.58***	***<0.001***	***<0.001***	***1.34***	***1.17-1.55***	***<0.001***	***0.001***
AURKA	1.34	1.06-1.7	0.013	0.038	1.13	0.83-1.56	0.437	0.557
BUB1	1.13	0.94-1.34	0.184	0.282	0.97	0.74-1.27	0.824	0.875
CXCL12	0.87	0.71-1.08	0.215	0.282	0.94	0.75-1.17	0.571	0.647
**KRT5**	**1.06**	**0.95-1.18**	**0.308**	**0.375**	**1.18**	**1.02-1.36**	**0.028**	**0.094**
**KRT6A**	**1.21**	**1.07-1.37**	**0.003**	**0.012**	**1.19**	**1.03-1.37**	**0.016**	**0.067**
**KRT6B**	**1.12**	**0.98-1.28**	**0.094**	**0.178**	**1.18**	**1.01-1.38**	**0.041**	**0.117**
EGFR	1.65	1.06-2.57	0.027	0.065	1.76	0.98-3.15	0.059	0.142
**MYBL2**	**1.56**	**1.23-1.97**	**<0.001**	**0.002**	**1.49**	**1.11-2.00**	**0.008**	***0.047***
NDRG1	1.14	0.99-1.32	0.062	0.132	1.12	0.95-1.32	0.185	0.342
MYCN	1.3	0.88-1.93	0.185	0.282	1.23	0.82-1.84	0.311	0.481
SNAI1	0.88	0.64-1.21	0.425	0.452	0.81	0.58-1.12	0.201	0.342
UGT8	0.97	0.8-1.17	0.726	0.726	0.89	0.7-1.14	0.368	0.522
**VEGFA**	**1.34**	**1.13-1.59**	**<0.001**	**0.005**	**1.28**	**1.07-1.53**	**0.007**	***0.047***
SIX1	1.07	0.96-1.18	0.213	0.282	1	0.9-1.13	0.939	0.939
EYA2	0.92	0.75-1.13	0.416	0.452	0.93	0.76-1.14	0.458	0.557

A Cox regression model was used to evaluate the association between the mRNA expression levels of each of the 17 candidate genes and DFS at univariate and multivariate levels. The multivariate model adjusted for tumor subtype and grade. The bold genes are significant at nominal 0.05 significance level and the bold italic genes are significant at 0.05 adjusted significance level in multivariate model.

### Association between gene expression and DFS for HER2-enriched and basal breast cancer subtypes in univariate and multivariate models

Recognizing the aggressive nature and increased incidence of HER2-enriched and basal breast cancer among younger women, we performed a similar analysis within these two breast cancer subtypes. Within the basal subtype, overexpression of *ANGPTL4* (HR 1.5, CI 1.17-1.96, unadjusted *p* = 0.002, adjusted *p* = 0.034) was significantly associated with DFS when correcting for grade in multivariable analysis (Table 5). There was a trend toward increased risk of disease recurrence for *KRT5* (HR 1.17, *p* = 0.072)*, KRT6A* (HR 1.17, *p* = 0.092), *EGFR* (HR 1.96, *p* = 0.072) and *VEGFA* (HR 1.29, *p* = 0.08). Interestingly, among older patients with basal breast cancer, expression levels of *ANGPTL4, KRT5, KRT6A, EGFR* and *VEGFA* were not associated with DFS. (Supp. Table 2).

**Table 5:** Association between expression levels and DFS in Basal-like group (Young group)

Gene Name	Univariate Model				Multivariate Model			
	Hazard Ratio	95% CI	*P* value	Adjusted *P* value	Hazard Ratio	95% CI	*P* value	Adjusted *P* value
ADM	1.2	0.98-1.47	0.081	0.438	1.21	0.99-1.47	0.062	0.261
***ANGPTL4***	***1.5***	***1.16-1.94***	***0.002***	***0.034***	***1.51***	***1.17-1.96***	***0.002***	***0.034***
AURKA	1.24	0.67-2.27	0.495	0.561	1.39	0.77-2.51	0.281	0.434
BUB1	0.85	0.58-1.24	0.389	0.477	0.91	0.62-1.36	0.659	0.747
CXCL12	1.05	0.8-1.39	0.731	0.751	1.05	0.8-1.39	0.719	0.764
KRT5	1.18	0.99-1.41	0.070	0.438	1.17	0.99-1.39	0.072	0.261
KRT6A	1.17	0.97-1.4	0.103	0.438	1.17	0.97-1.4	0.092	0.261
KRT6B	1.1	0.91-1.33	0.307	0.477	1.14	0.94-1.38	0.180	0.415
EGFR	1.79	0.8-4.02	0.159	0.450	1.96	0.94-4.08	0.072	0.261
MYBL2	1.27	0.79-2.02	0.323	0.477	1.3	0.83-2.03	0.244	0.415
NDRG1	1.14	0.91-1.42	0.264	0.477	1.14	0.92-1.41	0.232	0.415
MYCN	1.44	0.63-3.3	0.393	0.477	1.67	0.71-3.96	0.242	0.415
SNAI1	0.73	0.42-1.26	0.252	0.477	0.78	0.44-1.37	0.380	0.497
UGT8	0.87	0.64-1.16	0.341	0.477	0.89	0.66-1.19	0.422	0.512
VEGFA	1.24	0.93-1.65	0.149	0.450	1.29	0.97-1.71	0.080	0.261
SIX1	0.97	0.79-1.19	0.751	0.751	0.98	0.8-1.21	0.870	0.870
EYA2	0.79	0.55-1.13	0.192	0.466	0.83	0.56-1.21	0.324	0.459

The bold genes are significant at nominal 0.05 significance level and the bold italic genes are significant at 0.05 adjusted significance level in multivariate model.

The results for HER2-enriched subtype in young and old patient groups are shown in Table 6 and Supp. Table 3. In younger patients, there was an association between disease recurrence and overexpression of the following genes in a multivariate model correcting for grade*: KRT5* (HR 1.45, CI 1.01-2.07, *p* = 0.044), *KRT6A* (HR 1.73, CI 1.13-2.65, *p* = 0.012), *KRT6B* (HR 2.15, CI 1.29-3.59, *p* = 0.003), *MYBL2* (HR 1.72, CI 1.04-2.85, *p* = 0.035) and *SNAI1* (HR 0.51, CI 0.28-0.91, *p* = 0.023) (Table 6). Correction for multiple gene comparison showed that *KRT6B* showed a trend toward significance (*p* = 0.051). Interestingly, in the older group of patients, the only gene that maintained an association with DFS was *MYBL2* (HR 1.46, CI 1.05-2.02, *p* = 0.023) (Supp. Table 3).

**Table 6:** Association between expression levels and DFS in HER2 group (Young group)

Gene Name	Univariate Model				Multivariate Model			
	Hazard Ratio	95% CI	P value	Adjusted *P* value	Hazard Ratio	95% CI	*P* value	Adjusted *P* value
ADM	0.96	0.76-1.21	0.707	0.997	0.94	0.73-1.21	0.617	0.862
ANGPTL4	1.26	0.98-1.61	0.074	0.230	1.26	0.98-1.62	0.069	0.196
AURKA	0.85	0.46-1.57	0.602	0.930	0.86	0.47-1.58	0.619	0.862
BUB1	0.99	0.62-1.57	0.963	0.997	1.01	0.63-1.61	0.973	0.973
CXCL12	1.01	0.63-1.63	0.972	0.997	1.01	0.63-1.63	0.961	0.973
**KRT5**	**1.32**	**0.98-1.79**	**0.072**	**0.230**	**1.45**	**1.01-2.07**	**0.044**	**0.150**
**KRT6A**	**1.6**	**1.06-2.4**	**0.024**	**0.193**	**1.73**	**1.13-2.65**	**0.012**	**0.102**
**KRT6B**	**2.14**	**1.29-3.54**	**0.003**	**0.051**	**2.15**	**1.29-3.59**	**0.003**	***0.051***
EGFR	1	0.4-2.49	0.997	0.997	0.97	0.38-2.45	0.944	0.973
**MYBL2**	**1.51**	**0.95-2.4**	**0.081**	**0.230**	**1.72**	**1.04-2.85**	**0.035**	**0.149**
NDRG1	1.05	0.77-1.43	0.772	0.997	1.08	0.77-1.51	0.659	0.862
MYCN	0.99	0.59-1.68	0.978	0.997	1.04	0.58-1.88	0.894	0.973
**SNAI1**	**0.55**	**0.32-0.96**	**0.034**	**0.193**	**0.51**	**0.28-0.91**	**0.023**	**0.130**
UGT8	1.38	0.77-2.45	0.276	0.521	1.37	0.76-2.46	0.290	0.530
VEGFA	1.24	0.92-1.68	0.164	0.349	1.31	0.93-1.82	0.118	0.264
SIX1	0.84	0.68-1.05	0.119	0.289	0.84	0.68-1.05	0.124	0.264
EYA2	1.15	0.8-1.66	0.441	0.750	1.25	0.81-1.93	0.312	0.530

The bold genes are significant at nominal 0.05 significance level and the bold italic genes are significant at 0.05 adjusted significance level in multivariate model.

### Disease-free survival analysis using the Kaplan Meier method

To further interrogate the association of gene expression and DFS in basal and HER2-enriched subtypes known to frequently occur in young women, we categorized expression levels for each gene as high or low (cut at the median) and created Kaplan Meier survival plots based on high vs. low gene expression. For four genes in the data set, *ANGPTL4, KRT6A KRT6B* and *SNAI1*, there was a significant association between gene expression and DFS by the Kaplan Meier method in young women. None of the other genes were significant for the younger group.

**Figure 2:**
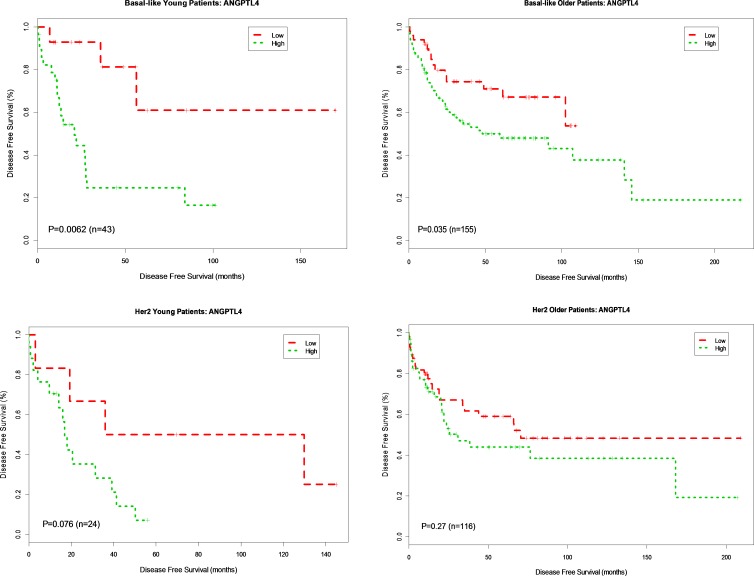
Kaplan-Meier DFS Curves for *ANGPTL4* Gene expression in Basal-like and Her2-enriched Breast Cancer Subtype Tumor samples were categorized into patients with high expression (green curve) and patients with low expression (red curve) based on *ANGPTL4* gene expression levels in 778 patients. DFS analysis between high expression and low expression patients was performed for young patients with basal-like, older patients with basal-like, young patients in Her2-enriched and older patient in Her2-enriched breast tumors, respectively.

**Figure 3:**
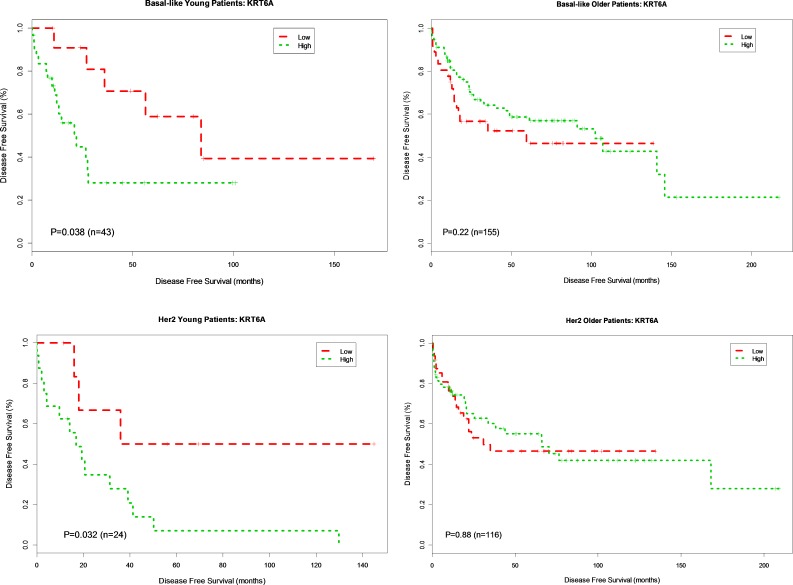
Kaplan-Meier DFS Curves for *KRT6A* Gene Expression in Basal-like and Her2-enriched Breast Cancer Subtype Tumor samples were categorized into patients with high expression (green curve) and patients with low expression (red curve) based on *KRT6A* gene expression levels in 778 patients. DFS analysis between high expression and low expression patients was performed for young patients with basal-like, older patients with basal-like, young patients in Her2-enriched and older patient in Her2-enriched breast tumors, respectively.

**Figure 4:**
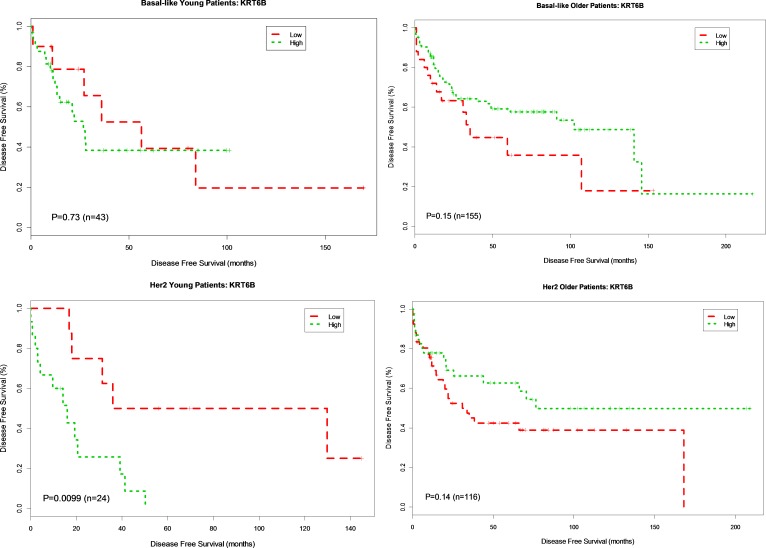
Kaplan-Meier DFS Curves for KRT6B Gene Expression in Basal-like and Her2-enriched Breast Cancer Subtype Tumor samples were categorized into patients with high expression (green curve) and patients with low expression (red curve). DFS analysis between high expression and low expression patients was performed for young patients with basal-like, older patients with basal-like, young patients in Her2-enriched and older patient in Her2-enriched breast tumors, respectively.

Overexpression of *ANGPTL4* was associated with worse DFS for both younger (*p* = 0.006, HR 4.76) and older patients (*p* = 0.035, HR 1.88) with basal breast cancer. This association was not seen in patients with HER2-enriched breast cancer (young, *p* = 0.076, HR 2.98; older *p* = 0.27, HR 1.37) (Figure 2). Overexpression of *KRT6A* was significantly associated with worse DFS in younger patients with both basal and HER2-enriched breast cancer (*p* = 0.038, HR 2.85; *p* = 0.032, HR 3.6, respectively). There was no association between *KRT6A* and DFS among older patients with either basal or HER2-enriched breast cancer (*p* = 0.22 and *p* = 0.88, respectively) (Figure 3). Overexpression of *KRT6B* was associated with worse DFS in younger patients with HER2-enriched breast cancer only (*p* = 0.01, HR 4.1); an inverse relationship was seen in older patients in this group (*p* = 0.14, HR 0.65) (Figure 4).

## DISCUSSION

In accordance with previous reports, our analysis reveals a higher frequency of high grade and endocrine insensitive breast tumors in young women as compared to older women, as well as age-related differences in the relative frequency of breast cancer subtypes by age [15], Specifically, we found an increased frequency of HER2-enriched and basal breast cancer subtypes in young as compared to older women. Despite similarities in receipt of systemic chemotherapy, young women with HER2-enriched and Luminal B breast cancer had inferior outcome compared to older women within the same subtype. We noted inferior survival for young women in the HER2-enriched subgroup, as well as a trend toward poor outcome in young women with basal and luminal B breast cancer. Based on the fact that patients in two of the three data sets (NKI295 and GSE4922) were diagnosed before 2003, it is likely that the majority of HER2-positive patients in this study were treated in the pre-Herceptin era [8] [9], and did not receive targeted therapy. It is possible that, with the advent of Herceptin, newer data sets may show less profound age-related differences. However, the baseline inferior survival of young women with HER2-positive disease is noteworthy. We also identified age-related differences in the expression of several key genes associated with proliferation, invasion and metastasis, some of which predicted inferior DFS in younger women. In univariate and multivariate modeling (accounting for subtype and grade), overexpression of *ANGPTL4, MYBL2* and *VEGF* were associated with inferior DFS for both the young and older age groups.

For three genes in the data set, *KRT5, KRT6A, KRT6B*, there was a significant association between gene expression and inferior prognosis unique to young women (with overexpression of *EGFR* of borderline significance). Overexpression of *ANGPTL4* was associated with inferior outcome for young women with basal breast tumors; the same held true for the keratins (*KRT5, KRT6A,* and *KRT6B*) among young women with HER2-enriched breast cancer. Kaplan-Meier survival analysis illustrates inferior DFS for young, but not older, patients with the HER2-enriched breast cancer over-expressing *KRT6A* and *KRT6B*. Taken together, this data suggests that the keratin genes may be involved in young adult cancers- beyond that of the basal subtype - and that overexpression of these genes may negatively impact outcome for women with young adult breast cancer. Finally, our analysis points toward *ANGPLT4* as a gene whose overexpression may be associated with poorer outcome among younger women with aggressive basal breast cancer.

Several previous studies have recently reported biological differences in the breast cancers of young women that extend beyond those attributable to age-related variation in subtype distribution. Using bacterial artificial chromosome (BAC) array comparative genomic hybridization (aCGH), Thomas and Leonard [6] identified preliminary evidence of predictable chromosomal copy number differences between grade 3, node negative breast tumors of young (< 45 years of age) vs. elderly women (>70 years of age). Benz (2008) [7] noted differences in invasiveness and angiogenesis in tumors of young vs. older women, suggesting age-related differences in epigenetic regulation. In 4,000 clinically-annotated breast cancer cases, those arising in older women were less aggressive and grew more slowly than those of younger women, even after controlling for both grade and expression of hormone receptors and HER2. Tumor protein extracts were analyzed by immunoassay for expression of 11 biomarkers selected to correlate with proliferation, angiogenesis, and endocrine dependence. Notably, while expression levels of uPA and VEGF, markers of angiogenesis and invasiveness, did not differ in an age-specific manner, the clinical impact of expression levels differed by age. For women with node-negative, ER-positive tumors, high expression levels of either of the two genes gene correlated with inferior DFS only for young patients < age 45, but not for older patients >70. This observation suggests an age-specific response among biologically similar tumors. Finally, Azim et al. [16] evaluated the prognostic significance of previously published gene signatures related to stroma, immunity and proliferation in breast cancers arising in young women (< 40 years of age) compared to older women. They found that stromal gene signatures had prognostic value only for young women with ER-negative, HER2-negative breast cancer, but not for older women, suggesting a role for tissue microenvironment in the pathogenesis of young adult breast cancer. Compared to breast cancers of older women, young adult breast cancers were relatively enriched for immature mammary cell populations and growth factor signaling, with relative downregulation of genes related to apoptosis. The authors concluded that these features of young adult breast cancers could potentially promote aggressive tumor growth. A difference in methodology between this study and ours is that, in this study, subtype was defined by a 3-gene classifier (ESR1, ERBB2 and AURKA), whereas our study classified tumors based on the PAM50.

The subgroup of “core basal” tumors overexpressing EGFR and cytokeratin 5/6 is particularly prevalent in young women under 40 as compared to older women. Previous studies have noted poor outcome associated with high expression of cytokeratin 5, 6A and 6B in basal tumors [5], but to our knowledge this is the first report that the association between cytokeratin expression and inferior DFS may be an age-related finding, present in young women with HER2-enriched as well as basal tumors, but not in older women with breast cancer. Thus, it is possible that high expression of cytokeratins 5 and 6 (CK 5/6) may be a more generalizable indicator of poor outcome in young breast cancer patients. CK 5/6 expression in primary breast tumors has previously been identified in association with the development of brain metastases or metastases at multiple sites [17]. Taken together, these data suggest the possibility that CK 5/6 may be involved in the clinically aggressive behavior of breast cancers in young adults. Interestingly, the same did not hold true for EGFR (another gene that defines the “core basal” subtype), which in our study had no impact on DFS in HER2-enriched breast cancers, but did approach significance for basal breast cancers in young women.

When corrected for subtype and grade, our multivariant analysis shows that high expression of ANGPTL4, a potential druggable target, strongly predicts inferior DFS in young but not older women with basal-type breast cancer. Kaplan Meier survival curves suggest an association between high expression of ANGPTL4 and inferior DFS for both the basal and HER2-enriched subtypes. While this is correlation is present in both the young and older age groups, it is more pronounced in the young patients. ANGPTL4 is a secreted matricellular protein that is broadly expressed in many types of malignant tumor and is associated with poor prognosis in oral cancer [18]. ANGPTL4 plays a critical role in cancer growth and progression and specifically contributes to breast cancer metastasis by protecting endothelial cells from apoptosis promoting angiogenesis, and facilitating cell migration [18] [19]. High expression of ANGPTL4 in primary breast tumors is strongly associated with metastasis to the lung and has also been implicated in brain metastasis in breast cancer [20] [21]. It is well-recognized that patterns of metastatic spread differ by breast cancer subtype, with the basal subtype highly prone to brain and lung metastases [22] [23]. The role of ANGPTL4 in breast cancer metastasis to both lung and brain makes it an interesting potential druggable target. As a direct target of HIF-1, ANGPTL4 is a candidate for clinical intervention using digoxin, which inhibits HIF-1 and has been shown to decrease tumor growth and lung metastasis breast cancer cell lines and xenografts [24] [25]. The use of either general angiogenesis inhibitors or specific agents against ANGPTL4 may prove particularly beneficial for young adult patients with basal breast cancer, a high risk population in need of more effective therapeutics.

We recognize that our study had several limitations. Survival analyses were impacted by the fact that the databases include limited information regarding the specifics of cancer therapy. Our survival analyses therefore, are exploratory in nature and will require validation in larger, population-based studies. Instead of a genome-wide exploratory analysis, we selected a smaller number of genes to analyze based on published reports suggesting a potential role in the development of breast cancer in young women. Focus on candidate genes identified through a literature search may decrease the potential for bias due to multiple testing that is inherent in comparative studies of global gene expression. We also recognize that there is no single best way to explore the biology of young women's breast tumors. In this study, we took a similar approach to Azim et al [16]. While our previous large scale analysis of gene expression did not reveal striking age-related differences [4], targeted analysis of genes relating to proliferation, invasion and metastasis within breast cancer subtypes suggests significant age-related differences in several key genes (i.e. ANGPTL4 and cytokeratins 5 and 6) that may hold prognostic significance.

## CONCLUSIONS

Taken together, these data are preliminary, yet provocative, and should be validated in future studies. If validated, this information may prove useful to young women and their physicians as they make treatment decisions for early stage and/or advanced breast cancer, especially as genotyping becomes more commonplace in clinical practice. Remaining unanswered questions include (1) the biological basis for the preponderance of aggressive subtypes of breast cancer arising in younger women and (2) the role of the microenvironment in the development of young adult breast cancers – both of which are research subjects worthy of further pursuit.

## MATERIALS AND METHODS

### Patient selection and breast carcinoma samples

Microarray data from three publically-available, clinically-annotated breast cancer data sets, NKI295 [8] (*n* = 259; normal-like tumors were excluded from the analysis), GSE4922 [9] (*n* = 205) and GSE20624 [4] (*n* = 314), were used for the analysis. At the time of this analysis, these datasets were selected based on (1) their inclusion of a substantial number of patients under the age of 40, (2) our ability to merge platforms to conduct the analysis, and (3) their inclusion of all 17 genes of interest on the respective platforms. NKI295 and GSE20624 data sets were generated by two-channel Agilent microarray while GSE4922 data was based on Affymetrix one-channel microarray. We used the normalized data from original studies, which has been row (gene) median centered and column (sample) standardized. Batch correction was performed on the three data sets (*n* = 778) using an empirical Bayes approach [10]. A total of 778 clinically-annotated breast tumor samples from the three data sets were available for analysis. All three data sets included information on age, breast cancer subtype, hormone receptor status (ER/PR), tumor size, tumor grade and nodal status. None of the data sets contained information about familial risk of breast cancer. Two of the three data sets (NKI295 and GSE20624) also contained information on treatment. (Table 1)

### Clinicopathological characteristics and breast cancer subtype assignment

The following clinicopathologic variables were available for analysis: ER status (positive/negative), tumor size (T ≤ 2 cm, T > 2cm), tumor grade (1, 2, 3), lymph node status (positive/negative), treatment (chemotherapy [yes/no], chemotherapy and endocrine therapy [yes/no], endocrine therapy only [yes/no], or no systemic therapy). In addition, the 50-gene Prediction Analysis of Microarray (PAM50) classifier was applied to the data and classified breast tumors as Luminal A (LumA), Luminal B (LumB), HER2-enriched, and basal [11]. For the purposes of this analysis, the Normal-like classification was not included. Fisher's exact test implemented in R (http://www.r-project.org/) was used to evaluate the association between each clinicopathological variable and the age groups (< 40 years and >=40 years). Two-tailed *P* value < 0.05 was considered to be statistically significant.

### Selection of candidate genes

We conducted a PubMed search for genes that are associated with poor outcome in breast cancer, focusing on genes implicated in breast cancer proliferation, invasion, metastasis or patient survival. Search terms included: breast cancer gene expression and metastasis, breast cancer gene expression and death, breast cancer and early onset. The 17 genes selected, along with their biologic functions, are listed in Table 2.

### Differential analysis of single gene expression between age groups

Patients were categorized into two groups: young (aged < 40 years) and older (aged ≥ 40) at breast cancer diagnosis. Age-specific differences in single-gene mRNA expression values were tested using linear regression models (*lm* function in R). The analysis was conducted at both univariate and multivariate levels for each gene. In the multivariate model, we adjusted for significant clinical variables to include tumor grade and tumor subtype (Table 1). Although the estrogen receptor was a significant clinical variable, this variable was not included in the multivariate model as it is known to correlate with breast cancer subtype. The corresponding p-values from univariate and multivariate models were adjusted using the multiple testing procedure developed by Benjamini and Hochberg [12].

### Survival analysis

We defined a disease-free survival (DFS) event as the time from diagnosis of breast cancer to either identification of disease recurrence or death, whichever occurred first. DFS was censored at last follow-up for those alive without recurrence. The Kaplan-Meier method was used for the DFS analysis and was performed for two categorical variables: (1) age (young defined as < 40 years or older defined as ≥ 40 years of age at breast cancer diagnosis) and (2) gene expression (high expression or low expression). High expression was defined as gene expression greater than the median expression value across all (*n* = 778) patients for a given gene, while low expression was defined as gene expression levels ≤ the median expression value across all (*n* = 778) patients for a given gene. The log-rank test was used to evaluate the association between the gene expression and patient survival [13]. More specifically, for (1), we performed the analysis within PAM50 subtypes; for (2), we performed the analysis for each gene by PAM50 subtypes and age, respectively.

A Cox regression model [14] was used to evaluate the association between the mRNA expression levels of each of the 17 candidate genes and DFS at univariate and multivariate levels. This analysis was performed across all subtypes as well as within the basal and HER2 subtypes by age, given that the tumors of young women are enriched for these subtypes [4]. For the analysis of the group, the multivariate model was adjusted for significant clinical variables: tumor grade and PAM50 tumor subtype, as previously described. For the analysis within subtypes, the multivariate model was adjusted for tumor grade only. The corresponding p-values from univariate and multivariate models were adjusted using the multiple testing procedure developed by Benjamini and Hochberg [12]. All analyses were performed using Survival R package (http://cran.r-project.org/web/packages/survival/).

## SUPPLEMENTARY MATERIALS, TABLES



## Abbreviations

aCGH: array comparative genomic hybridization
BAC: bacterial artificial chromosome
CK: cytokeratin
DFS: disease-free survival
EGFR: epithelial growth factor receptor
ER: estrogen receptor
HER2: human epidermal growth factor receptor 2
LumA: Luminal A
LumB: Luminal B
PAM50: Prediction Analysis of Microarray
PR: progesterone receptor.
