# Supplementary material for: Gene expression in “young adult type” breast cancer: a retrospective analysis
Source: Oncotarget. 2015 May 9;6(15):13688–702. doi: 10.18632/oncotarget.4051 (PMC4537042; doi:10.18632/oncotarget.4051)
Supplement: Supplementary file 1 [file oncotarget-06-13688-s001.pdf]

Gene expression in "young adult type" breast cancer: a retrospective analysis

Supplementary Material

Supplemental table 1: Association between expression levels and DFS in all subtypes (Older group)

The bold genes are significant at nominal 0.05 significance level and the bold italic genes are significant at 0.05 adjusted significance level in multivariate model.

Gene Name	Univariate Model				Multivariate Model			
	Hazard Ratio	95% CI	*P* value	Adjusted *P* value	Hazard Ratio	95% CI	*P* value	Adjusted *P* value
ADM	***1.17***	***1.1-1.26***	***<0.001***	***<0.001***	***1.11***	***1.03-1.19***	***0.009***	***0.049***
**ANGPTL4**	**1.10**	**1.02-1.18**	**0.001**	**0.021**	**1.08**	**1.00-1.16**	**0.048**	**0.162**
AURKA	1.39	1.25-1.54	<0.001	<0.001	1.15	0.99-1.33	0.065	0.172
BUB1	1.29	1.18-1.42	<0.001	<0.001	1.02	0.88-1.19	0.778	0.945
CXCL12	0.88	0.8-0.96	<0.001	0.018	0.96	0.87-1.07	0.501	0.774
KRT5	0.98	0.92-1.04	0.521	0.553	0.99	0.92-1.07	0.854	0.968
KRT6A	1.06	0.98-1.16	0.145	0.176	1	0.90-1.10	0.929	0.987
KRT6B	0.99	0.92-1.07	0.792	0.792	0.95	0.87-1.05	0.321	0.622
EGFR	1.18	1.04-1.35	0.012	0.022	1.08	0.93-1.25	0.329	0.622
MYBL2	***1.50***	***1.34-1.69***	***<0.001***	***<0.001***	***1.28***	***1.10-1.50***	***0.002***	***0.030***
NDRG1	***1.20***	***1.11-1.3***	***<0.001***	***<0.001***	***1.14***	***1.04-1.25***	***0.005***	***0.042***
MYCN	1.13	0.86-1.49	0.382	0.432	1.07	0.80-1.43	0.655	0.927
SNAI1	1.16	1.03-1.31	0.013	0.022	1.13	0.99-1.28	0.071	0.172
UGT8	1.09	0.99-1.21	0.086	0.130	1.02	0.88-1.18	0.774	0.945
**VEGFA**	**1.23**	**1.11-1.36**	**<0.001**	**<0.001**	**1.14**	**1.01-1.28**	**0.033**	**0.142**
SIX1	1.04	0.99-1.1	0.127	0.166	1.00	0.94-1.06	0.998	0.998
EYA2	1.10	0.98-1.24	0.092	0.130	1.05	0.94-1.18	0.372	0.632

Supplemental table 2: Association between expression levels and DFS in basal group (Older group)

The bold genes are significant at nominal 0.05 significance level and the bold italic genes are significant at 0.05 adjusted significance level in multivariate model.

Gene Name	Univariate Model				Multivariate Model			
	Hazard Ratio	95% CI	*P* value	Adjusted *P* value	Hazard Ratio	95% CI	*P* value	Adjusted *P* value
ADM	1.13	0.99-1.3	0.074	0.504	1.14	0.99-1.32	0.063	0.436
ANGPTL4	1.05	0.93-1.19	0.441	0.833	1.06	0.94-1.21	0.339	0.735
AURKA	0.98	0.71-1.34	0.893	0.899	0.94	0.67-1.32	0.731	0.956
BUB1	1.04	0.79-1.37	0.780	0.899	0.99	0.73-1.33	0.924	0.967
CXCL12	0.88	0.71-1.07	0.200	0.554	0.9	0.73-1.12	0.346	0.735
KRT5	1.01	0.91-1.13	0.823	0.899	1.03	0.92-1.15	0.577	0.956
KRT6A	0.96	0.83-1.11	0.557	0.861	0.98	0.85-1.13	0.793	0.963
KRT6B	0.96	0.86-1.09	0.547	0.861	0.99	0.88-1.12	0.893	0.967
EGFR	1.17	0.91-1.5	0.228	0.554	1.13	0.85-1.48	0.401	0.757
MYBL2	1.28	0.88-1.87	0.189	0.554	1.34	0.91-1.96	0.137	0.582
NDRG1	1.06	0.92-1.23	0.419	0.833	1.09	0.94-1.26	0.271	0.735
**MYCN**	**0.52**	**0.29-0.93**	**0.028**	**0.476**	**0.49**	**0.27-0.89**	**0.019**	**0.323**
SNAI1	1.2	0.9-1.62	0.217	0.554	1.23	0.91-1.67	0.180	0.612
UGT8	1.01	0.84-1.23	0.877	0.899	1.04	0.85-1.26	0.718	0.956
VEGFA	1.03	0.85-1.26	0.750	0.899	1.05	0.86-1.29	0.632	0.956
SIX1	1.01	0.88-1.15	0.899	0.899	1	0.87-1.14	0.967	0.967
EYA2	1.2	0.97-1.49	0.089	0.504	1.22	0.98-1.52	0.077	0.436

Supplemental table 3: Association between expression levels and DFS in HER2 group (Older group)

The bold genes are significant at nominal 0.05 significance level and the bold italic genes are significant at 0.05 adjusted significance level in multivariate model.

Gene Name	Univariate Model				Multivariate Model			
	Hazard Ratio	95% CI	*P* value	Adjusted *P* value	Hazard Ratio	95% CI	*P* value	Adjusted *P* value
ADM	1.08	0.94-1.24	0.274	0.580	1.09	0.94-1.25	0.253	0.570
ANGPTL4	1.15	0.99-1.35	0.077	0.218	1.15	0.99-1.35	0.074	0.210
**AURKA**	**1.29**	**1.01-1.65**	**0.040**	**0.167**	**1.3**	**1.02-1.65**	**0.035**	**0.149**
BUB1	0.97	0.71-1.33	0.862	0.973	0.98	0.72-1.34	0.905	0.976
CXCL12	1.03	0.83-1.29	0.778	0.945	1.03	0.81-1.29	0.834	0.976
KRT5	0.92	0.78-1.09	0.335	0.580	0.91	0.76-1.08	0.283	0.570
KRT6A	0.94	0.77-1.14	0.523	0.741	0.94	0.77-1.14	0.524	0.742
KRT6B	0.89	0.7-1.13	0.336	0.580	0.89	0.7-1.13	0.335	0.570
EGFR	1.12	0.87-1.44	0.395	0.610	1.12	0.86-1.44	0.400	0.618
**MYBL2**	**1.45**	**1.05-2**	**0.025**	**0.167**	**1.46**	**1.05-2.02**	**0.023**	**0.142**
**NDRG1**	**1.23**	**1.02-1.49**	**0.032**	**0.167**	**1.25**	**1.03-1.52**	**0.025**	**0.142**
**MYCN**	**2.02**	**1.24-3.29**	**0.005**	**0.085**	**2.02**	**1.23-3.3**	**0.005**	**0.085**
SNAI1	0.99	0.74-1.34	0.973	0.973	1	0.74-1.34	0.979	0.979
**UGT8**	**0.63**	**0.4-1**	**0.049**	**0.167**	**0.63**	**0.4-0.99**	**0.046**	**0.156**
VEGFA	1.13	0.88-1.44	0.341	0.580	1.13	0.88-1.45	0.329	0.570
SIX1	1.02	0.9-1.17	0.737	0.945	1.02	0.9-1.17	0.734	0.960
EYA2	0.99	0.78-1.25	0.936	0.973	0.99	0.78-1.25	0.919	0.976
